# Online mental health services during COVID-19 pandemic in Indonesia: Challenges from psychologist perspective

**DOI:** 10.1371/journal.pone.0285490

**Published:** 2023-06-23

**Authors:** Alma Marikka Geraldina, Mein-Woei Suen, Passakorn Suanrueang

**Affiliations:** 1 Department of Healthcare Administration Specialty in Psychology, College of Medical and Health Science, Asia University, Taichung, Taiwan; 2 Department of Psychology, Asia University, Taichung, Taiwan; 3 Faculty of Psychology, Universitas Sebelas Maret, Surakarta, Indonesia; 4 Gender Equality Education and Research Center, Asia University, Taichung, Taiwan; 5 Department of Medical Research, Asia University Hospital, Asia University, Taichung, Taiwan; 6 Department of Medical Research, China Medical University Hospital, China Medical University, Taichung, Taiwan; School of Health Binawan: Universitas Binawan, INDONESIA

## Abstract

COVID-19 are causing many psychological impacts and change many aspects of human life. Mental health services also experiencing changes because of COVID-19 outbreak. In Indonesia, COVID-19 outbreak prompted the rapid development of online mental health services. These online mental health services which will help people to connect with professional mental healthcare providers using technology were created in response to this pandemic. Therefore, converting mental health services into online services in a state of urgency is challenging. This qualitative case study aims to provide a map of the service challenges that professional healthcare providers face while providing online services at present time by interviewing eight Indonesian psychologists as one of the mental health professionals who provided the online mental health services. Semi-structured interviews were done using interview guidelines with open-ended questions, and any other complementary data was collected using questionnaire. The data gathered from interviews was first performed through triangulation and then analyzed using thematic network analysis, which resulted in the following challenges: (1) building engagement; (2) risk of getting distracted during the sessions; (3) maintaining professional boundaries during the sessions; (4) keeping the personal information and confidentiality of the sessions; (5) perceived efficacy; and (6) attitudes towards online sessions. According to the results, the relevant organization can use this finding to contribute and develop the online mental health services both in this COVID-19 situation and in the future.

## Introduction

Since the first case of Corona Virus Disease 2019 (COVID-19) in Indonesia appeared on March 2, 2020, Indonesian government urged citizens in several cities to restrict activities outside their homes, do social distancing, avoid public gatherings such as religious events, weddings, and close some public places like schools and offices in the same month as the first cases appear [1]. The changes of situations because of COVID-19 pandemic then causing change to every aspects of human life, including people psychological condition.

World Health Organization (2020) reported that the prevalence of depression and anxiety symptoms due to COVID-19 were increasing. In America, 33% of adults were experienced symptoms of depression and 45% were experienced stress or excessive stress [2]. In Indonesia, 153 clients who accessed online consultation provided by psychology consultation unit of Gadjah Mada University suffered of psychological problems because of COVID-19 pandemic. These clients mostly complained about anxiety and depressive symptoms related to the public news about the spread of the virus. Many of them also felt stressed because of various problems such as losing jobs, boredom, and the rise of family problem because the increasing frequency of interaction between family members [3].

Study conducted by Serafini et al. (2020), shows the core psychological impact experienced in the general population is the decrease in social well-being. This decrease was caused by specific and uncontrolled fear, pervasive anxiety, frustration and boredom, and loneliness. If that condition is supported by inadequate social support and resilience, it can lead people to psychiatric conditions [4]. These psychological problems caused by COVID-19 pandemic and the increasing number of these mental health problem cases shows that the need of mental health services are also increasing.

In response to the increasing need of mental health services, these services were started to be offered online by several people who works in the mental health services field as a form of humanity [5]. Similar condition also exists in Indonesia. In Indonesia, the invention of technology in mental health services is still in very initial stages before COVID-19 pandemic forcing the rapid development of these online services [5, 6]. During COVID-19, several online mental health services and platforms were created as response to this pandemic. These platforms will help to connect people with professional mental healthcare providers. Furthermore, many institution such as psychological center, hospital, and other places which usually offers mental health services started to offer online services.

Mental health services can be defined as direct and indirect care to patients with mental health problems in ambulatory settings. Direct services include diagnostic and problem evaluation, intervention, psychotherapy, counselling, prescription, and post-hospital care; Indirect services consist of some consultative and collaborative work with the community or organization. Mental health services are usually provided by mental health professionals such as psychiatrists, psychiatric nurses, psychiatrists, social workers, or psychologists. Therefore in some other settings, the services can be provided by primary health care providers trained in mental health skills [7].

In the current situation, mental health services are experiencing changes because of the COVID-19 outbreak, which prompted the rapid development of online mental health services in Indonesia. Online mental health services are a conveyance of psychological services like counselling and psychotherapy where the psychologist and the client are not within the same physical area and use technology such as messaging, telephone, or video conferencing to communicate [5, 8].

On April 29, 2020, Indonesian government invented mental health services called *SEJIWA* with the cooperation of the Indonesian Ministry of Women Empowerment and Child Protection, Presidential Office, Ministry of Health, Ministry of Communication and Informatics, and Indonesian National Disaster Management Authority, and Indonesian Psychological Association. This service is providing education, counselling, and psychological services by contacting the call center 119 [9]. In addition to the service provided by the government, several private online mental health services and platforms were created. These platforms were aimed to help people to connect with professional mental healthcare providers by chat, telephone, or video conference [10].

On the other hand, the rapid development of online mental health services as a new transformation are followed by many challenges to face. Limited study were founded studying these transformation. Study results from Békés et al., (2021) explains that psychotherapist as one of the mental health providers are struggling with emotionally connected with clients, being distracted in the middle of the sessions, keeping patient’s privacy, and maintaining their boundaries in sessions [6]. Therefore, no study that mapped the challenges of Indonesian mental health services such as psychologists was made. Thus, this study wants to do mapping about online mental health services in Indonesia, and the challenges they face in the present time, and in the future by interviewing some Indonesian psychologists as one the mental health professionals who provided online mental health services.

The core research questions of this study is: what challenges that psychologist faces in providing online mental health services? This study is expected to pinpoint the challenges faced by these professionals and contribute to the development of online mental health services provided by psychologists both in this COVID-19 condition and in the future.

## Materials & method

### Research design & participants

This research is a qualitative case study research. Stake’s singe instrumental case study design were adopted for this study. This approach allows a comprehensive understanding of a phenomenon within real-life environment from the perspective of the people involved [11]. This research wants to map and explore challenges encountered while providing online mental heal services in real world settings. Stake’s methodology was adopted because the methodology provide the flexibility to begin with an unstructured conceptual framework which will be suitable with the research purposes.

Data for this research were gathered using a semi-structured interview with open-ended questions. The multidisciplinary research team who conducted this research (AM, PS, SM) were experienced in doing qualitative research, had educational background in psychology and public health, has training experience in mental health services, and participated in providing mental health services.

This study was involving psychologists as professional mental health providers as participants, which was chosen using snowball sampling. Participants were recruited from small initial contact of the first author (AM) who meets the inclusion criteria for participants: (1) Indonesian psychologist; (2) licensed clinicians; (3) providing online mental health services for at least 3 months; (4) had no experience providing online services before COVID-19 pandemic; then asks recommendation from participants recruited to recruit more participants.

Potential participants then given informed consent and a demographic data questionnaire using Google form. Demographic data gathered for this research are consist of personal information, then several questions which contain important information related to background education to know the major field the participants focused in providing the service. Location and healthcare settings to see where the online mental health services are mostly provided. Licensure status data to prove the participants are certified psychologist. Years of clinical experience were gathered to anticipate probability of years of experience influence. Previous experience on providing online services are needed to make sure participants which participated in this research, have enough experience in providing the service. Training in providing online services to anticipate the differences between those who got trained and have not get trained yet. Finally, what kind of online service provided data are gathered to have information about the form of service can which offered.

Twenty five participants were contacted, twelve participants were agree to participate in this research and filling out the initial questionnaire containing demographic data and informed consent, but eight participants then being chosen to participate. The rest of participants (four people) were excluded from the study because does not have enough experience in online services (less than three months of experience). Demographic data of participants in this study are explained in Table 1.

**Table 1:** Demographic characteristics of study participants. This table shows demographic data of eight participants participated in this study. Starts from personal information until mental health service provided.

	n	%
**Age**		
40–50	1	12.5
30–40	3	37.5
20–30	4	50
**Gender**		
Male	3	37.5
Female	5	62.5
**Educational Background**		
Clinical psychologist	4	50
Industrial and organizational psychologist	2	25
School and developmental psychologist	2	25
**Location**		
Jakarta	4	50
Surabaya	2	25
Yogyakarta	2	25
**Licensure status**		
Licensed	8	100
Trainee	0	0
**Years of clinical experience**		
1–2 years	3	37.5
3–4 years	2	25
>5 years	3	37.5
**Healthcare settings**		
Hospital/clinics	2	25
Psychology center	4	50
Mental health platform	2	25
**Previous experience on providing online services**		
>1 years	2	25
1–2 years	6	75
**Online services provided**		
Counselling	4	50
Counseling & psychotherapy	4	50
**Online services media**		
Message/chat, call, video conference	4	50
Call, video conference	4	50
**Previous training in providing online services**		
Yes	5	62.5
No	3	37.5

Eight chosen participants then participated in semi-structured interview sessions 1-to-1 with AM using video conference through zoom meetings. Before the interview was held, participants were given a written informed consent using Google Form which consist of information related to the topic being asked, purpose of the data gathering, process of data gathering, and contact person information. Participants which willing to participate filled out demographic data questionnaire and involved in at least one time online interview session lasted around one hour. These information also followed by agreement that any personal information gathered will be kept confidential. The interview process will be recorded in the form of video recording and will be kept confidential.

Participants which agree with the informed consent then being asked for submitting digital sign in the form as the proof of agreement to participate in the research. In the beginning of interview, participants were given explanation once again. Main question asked to the participant is: during the pandemic, what are the challenges you need to face while providing online mental health services?

Deeper questions then follows the main questions based on interview guideline. Interview guideline used in this research was adapted and developed from Békés et al. (2021) which mentioned that online mental health providers have several challenges to face, such as: (1) being emotionally connected with clients; (2) distracted in the middle of the sessions; (3) kept patient’s privacy; and (4) maintained their boundaries in sessions [6].

This interview guideline was made in Indonesian language then followed up with a content validation process through expert judgement and some changes were made as improvement. The interview guideline then were going through pilot testing stages by being asked to two Indonesian psychologist, to ensure that the interview guideline functions properly. The whole interview process was held using Indonesian language and being recorded for taking emotional notes, triangulation, and data analysis process later.

This research has been reviewed and approved by Health Research Ethics Committee Dr. Moewardi General Hospital, Indonesia (file number: 1.424./XI/HREC/2022). All participants in this study were recruited voluntarily and given informed consent with following rights as participants to freely participate in this study. All the data gathered are being kept confidential and anonymous.

### Data analysis

Data gathered from the interview are analyzed using thematic network analysis which consists of several steps: (1) coding the material; (2) identifying themes; (3) constructing a thematic network; (4) describing and exploring the thematic network; (5) summarize the thematic network; (6) interpret patterns. This analysis was started by mentioning the codes, identifying the issues, identifying themes, organizing the themes, and deducting into global themes [12]. The thematic analysis process was done with the assist of data management software Nvivo 11 and this manuscript are following Consolidated Criteria for Reporting Qualitative Research (COREQ) checklist.

During interview, field notes were taken and complete conversation transcript was made over the recording by the end of the interviews. The transcript then being returned to the participants for clarification, more explanation, comments. Afterwards, the data being translated into English separately by AM and professional translator. Any differences are being discussed,

Two different investigators (AM and PS) then continuing the process of data analysis which is identifying the codes and theme findings as a part of investigator triangulation, a validity check for data results. Any differences are being discussed between investigators, then data saturation were discussed between AM, PS, and MW. The final findings drafts then being presented to the participants to obtain any feedbacks.

## Results and discussion

A total of 10 interviews were completed, once interview per participant and two additional follow-up interviews, with the average for one and a half hour per interview. No new themes generated after these interviews which indicates the data had reached a saturation point. All participants participated in this study are licensed psychologist which offers counselling and psychotherapy as the form of mental health services using messaging (chat), call and video conference. Half of the participants have clinical psychology background, two of them have industrial and organizational psychology background, and the rest 2 of the participants are school and developmental psychologist. It can be referred from demographic data that online mental health services so far, are being centered in big cities in Indonesia such as Jakarta, Surabaya, and Yogyakarta, even though their clients could be from all over Indonesia or even Indonesian who lived abroad. These psychologist are offering the services under hospital/clinics, psychology center/bureau, and online mental health platform. In term of years of clinical experience, 37.5% of the participants have more than 5 years clinical experience, 25% of them have 3–4 years of clinical practice and the rest has lower than 2 years of clinical experience. Therefore, in term of providing online services, 62.5% of the participants has experience in doing online services for 1–2 years when the pandemic starts, then the rest of participants has experience less than a year. In addition, these demographic data also shown the lack of training for providing online mental health services, because only 37.5% of the participants had trained in providing online services.

Online mental health services challenge as the main focus of this research were determined before the data collected because the pandemic situations which causing obvious rapid development of online mental health services in Indonesia. Furthermore, after finished the interview, themes as the findings were identified based on the participants answers. As expected from qualitative research using semi-structured interview, main findings about online mental health services challenge were founded, then additional findings related to situation and culture challenges were added as complementary information.

### Online mental health challenges findings

**Fig 1:**
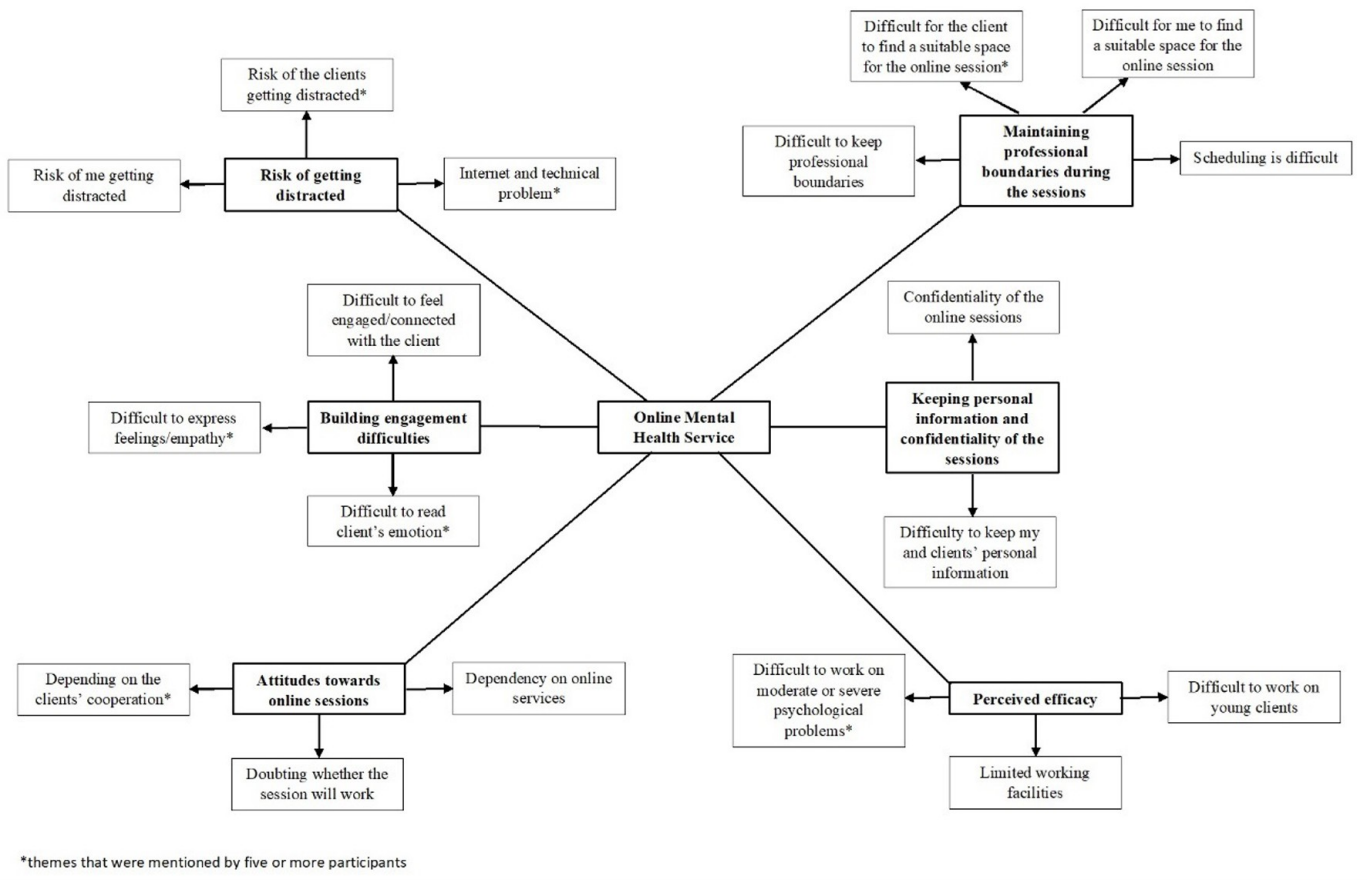
Illustration of mental health challenges findings. This figure illustrate the entire concept of findings started from themes and organized themes as challenges founded in this research.

The thematic network analysis method used in this data analysis step produced six global themes as the results. These themes are the challenges faced by a psychologist when providing online mental health services in Indonesia, which are: (1) building engagement difficulties; (2) risk of getting distracted; (3) maintaining professional boundaries during the sessions; (4) keeping personal information and confidentiality of the sessions; (5) perceived efficacy; (6) attitudes towards online sessions. An illustrations of finding themes can be seen in Fig 1.

#### Building engagement difficulties

Building engagement in online sessions is challenging because if the sessions are using message/chat or phone calls, psychologists are only focusing on the story, voice, and tone, and are unable to see the facial expression of the clients.

*“In the process of building rapport*, *it is much more difficult*, *especially in the form of a phone call that cannot be observed*, *it means we only focus on the voice*, *story*, *and intonation*, *which means that understanding people’s information is much more difficult than I met them in person*.*” (P1)*

Even when using video conference which allows psychologists to see client’s facial expressions, reading emotion still needs more effort from a psychologist because of face position, or the other details such as gestures, hand activity, and the other important gestures.

*“The observations are only the face*, *no hands*, *no legs*, *and nobody posture*. *Because it’s just his face*. *If we are offline*, *we can know*, *for example*, *if there is an indication of anxiety*, *the hand must be doing something*. *His hands were either clutching the table or the pillow*, *or the shirt*. *We can’t observe that*. *We don’t know whether his legs are moving*. *Or squeezing for people who are holding back the story*, *their hands are really clenched into fists*. *Squeezing his clothes for example” (P5)*

Similar to difficulties in reading emotions, delivering or expressing empathy to the client’s also challenging for the psychologist. It is because psychologists only can express their feelings with words. Thus, more verbal strategies are involved in expressing empathy to the clients.

*“The other obstacle is less effective in comforting her*. *Usually*, *I can show gestures that show empathy*, *so she’s more comfortable*, *for example*, *if a woman*, *I can hold her hand*, *I can pat her*, *that’s usually comforting for the client*. *But if you’re online*, *you can’t do things like that*. *It’s just voice and it’s also limited for comforting them” (P4)*

Based on these difficulties to read and expressing emotions to each other, feeling connected also becomes the challenge of the online sessions because psychologists cannot see the expressions and details of the clients. In addition, due to limited time, psychologists do not have time to really into building rapport and build a connection with the clients but focus directly on the problem of the clients

*“When analyzing the problem*, *we can’t help but generalize the client’s problems*, *it should be personalized*, *but because of this short rapport building we don’t really explore the client’s character*, *in the end*, *we just focus on the problem*, *and how to solve it*. *The building rapport is just left as it is” (P3)*

These themes were identified and then categorized into three organized themes which are: (1) difficult to read client’s emotion; (2) difficult to express feelings/empathy; (3) difficult to feel engaged/connected with the client. These three themes then being part of the building engagement challenge.

#### Risk of getting distracted

Online mental health services which always involve technology in the process, cannot be separated from interned and technical problems. One of these internet problems is freezing in the middle of the session, thus the conversation cannot be heard completely. The other internet problems are internet disconnection which makes the clients out in the middle of the sessions, a bad connection which causes lagging, and the application are stop working in the middle of the sessions. These problems are challenging because psychologists need to repeat and make sure both clients and their statements also start again if something makes the session disrupted.

*“Because this is online there is latency*, *yes connection problems*, *but this internet problem is still a pretty important problem*. *Inevitably it will affect how the session goes and it feels comfortable for the client because there is a lot of feedback from the client*, *some say there was a connection problem earlier so there are some things I have to make sure again I have to ask the psychologist again*, *or they say the psychologist video is a bit lagging*. *It’s really disturbing because the emotions are already established*, *the client is open*, *suddenly it’s “deg” (stop) like that*, *and sometimes the client is thrown off from the platform because of the connection*. *When he got the right moment*, *he was able to release his emotions*, *he was also in the phase to start a discussion*, *suddenly he is out when he came back the moment was gone*, *it was very distracting” (P2)*

Online mental health services which do not require a meeting between psychologists and the clients can be accessed from many places. In contrast to the accessibility, improper place for the sessions can also be distracting the sessions. An example is uncontrol conditions around the clients which can distract the sessions such as doing the session in the café which has live music. It can be distracting the psychologist’s concentration.

*“There are some people who take place in the cafe*, *it’s automatically noisy right*, *I’m disturbed too*, *right*, *I’m still focused*, *I need to take care of their feelings*, *I try to be careful with my sentences while the noise is really annoying*. *We can only remind them” (P1)*

In several cases, both the clients and the psychologist are sometimes also surrounded by people which can distract them from the sessions like family members are coming into their room.

*“I used to give her relaxation because she really needed it but suddenly her mom came in and she opened her eyes "Wait a minute*, *Mom*! *Close again*!*” even though it was a very deep session*. *That’s very annoying” (P5)*

These challenges psychologist faces can be categorized into three organizing themes which are: (1) internet problem and technical problem; (2) risk of me getting distracted; and (3) risk of the clients getting distracted. These challenges are included the risk of getting distracted challenge while providing online services.

#### Maintaining professional boundaries during the sessions

Online sessions involving technology could not be separated from a technical problem mentioned above. Therefore, psychologists need to deal with the possibility of any technical problems which happen during the sessions. In some cases, psychologists need to be patient while facing this technical problem even though it is exhausting for them.

*“Honestly*, *for me personally online is more tiring*, *maybe one of them because before starting an online session*, *there was a concern about internet problems*, *either on my internet or the client’s internet*. *It means that if the session is online*, *there are clients from remote areas as well*, *whose internet stability is not good*. *And we can’t say sorry*, *Ms*. *the network is having problems*, *even though we are the ones who are upset that the network is bad*. *So sometimes being patient with technical things is tiring” (P1)*

Psychologists as human beings need to regulate their emotions in online sessions to keep them professional. Therefore, because sometimes they are not able to control situations such as improper places of clients such as doing the sessions in the café which has noise from the activities around the clients, doing the sessions on the road, people pass by, psychologists need to deal with their own emotions regarding this condition. In addition, these conditions also indicate the difficulties of the clients to find a conducive place to do online sessions. This makes the sessions cannot be professional as expected

*“It sometimes happens in the beginning*, *counseling in a public place*. *And I need to control my emotions because we are still focused and there are noisy people which are very annoying*. *While I input information and I have to verbalize it again and I need concentration to organize it” (P6)*

These personal space issues also happen to the psychologist as the professional. Sometimes their place is not supportive for an online session, for example, no internet connection or the internet connection is bad because of raining hard.

Besides the challenge to keep professional boundaries and finding personal space for the sessions, commitment scheduling is also quite challenging for the online sessions. Before the sessions started, clients are given informed consent which includes information on what they should do or what they should prepare for the sessions. Therefore, many clients do not do what is written on the informed consent such as doing the session while lying down on the bed.

*“We always remind that counseling sessions must be followed properly*, *some clients are counseling while lying down*, *rolling around*, *smoking cigarettes*, *and some are eating and holding a spoon*. *There is a client who does the counseling while in the kitchen peeling onions*. *There is someone lying on the bed*, *her cellphone is lifted*, *and the camera is moving here and there*. *This distracts me*, *the session also becomes ineffective because looking at the screen rotates things*, *it’s making me dizzy*. *Difficult to condition*. *And when I remind her*, *she sat but lay down again*. *Sometimes their clothes are not proper*, *it’s too revealing*. *It’s indeed at home but that doesn’t mean you wear clothes for sleeping*. *I’m thinking can this session be serious for discussing the problem” (P2)*

In addition, clients which choose to do the sessions with chat sometimes are not staying on the sessions as scheduled. In some cases, after telling their condition to the psychologist and getting a reply, these clients do not give any reply anymore until the sessions are finished.

*“Usually*, *the client using chat is a bit hectic because the time is limited to one hour*, *one and a half*, *or two hours*. *Well*, *usually someone typed a long story straight away*, *so the psychologist needs to understand*, *right*, *read it first*, *digest it first*, *and complaints from the long story sometimes when we finish and give a response the client doesn’t respond anymore*, *doesn’t reply” (P7)*

These challenges identified are categorized into four organized themes, which are: (1) difficult to keep professional boundaries; (2) difficult for the client to find a suitable space for the online session; (3) difficult for me to find a suitable space for the online session; (4) scheduling is difficult. These challenges can be categorized as maintaining professional boundaries during the sessions.

#### Keeping personal information and confidentiality of the sessions

The next challenge, keeping confidentiality and personal information during psychological sessions is an important thing to be concerned about. Psychologists as professionals, in this case, understand how to keep the information given to them, but there are some conditions which out of psychologist controls. An example is clients who live with their families and do the sessions at home. Their family maybe can hear the things that happen during the sessions.

*“If it’s me*, *I’ll take good care of the data*, *but if he’s talking about his mother during the session and his mother can hear it*, *I don’t know*, *I can’t control it either*. *It’s more conditioned if it’s offline” (P8)*

In addition, sometimes online sessions are involving people which have responsibilities to handle technical problems such as IT staff. Therefore, involving more people which not aware of keeping the confidentiality of the sessions such as IT staff is quite risky.

*“Application for video conferencing we used Zoom*. *Sometimes if there’s something wrong*, *the admin staff has to be on standby*, *so there are 3 people involved*, *right*. *So*, *if there’s anything and the admin is not on standby*, *it will be stuck*. *And the psychologist doesn’t have the client’s numbers because it will be handled by the office*, *so there is really a third party*. *If one day there is a system error*, *we have to contact the IT person as the admin*. *Well*, *like it or not*, *the IT person will know about client data*. *So*, *in the clinical setting*, *many people are involved and those are not psychological people*. *So much riskier*.*” (P5)*

While providing online services, a psychologist is using their full name, personal accounts in their application, or personal social media. In offline settings, psychologists always say their full names but the clients usually just remember the nicknames. In contrast, in online settings this full name can easily be remembered by the clients because it is written, then in several chances, these clients are trying to see more personal information about their psychologist which means the personal information is more accessible during online sessions.

*“If we go online*, *our names will be listed on Zoom*. *Well*, *one day I saw on my “linked in that my profile was seen by my client*. *So*, *it’s more accessible*, *right*? *If you’re in the room*, *just your full name and nickname*, *but what they remember is just your nickname*. *But yes*, *some people from online session saw my linked-in*, *my Instagram” (P5)*

This challenge identified can be categorized into two organized themes which are: (1) confidentiality of the online sessions; and (2) difficulty to keep my and clients’ personal information. These two organized themes then being categorized into keeping the personal information and confidentiality of the sessions challenging.

#### Perceived efficacy

Online mental health services provided during this pandemic in Indonesia might be a good choice in response to this pandemic condition. However, psychologists find it difficult to use the online services if it is coming for moderate or severe psychological problems which makes the clients could not use their cognitive processing, and clients with an emergency conditions such as suicidal thoughts. The psychologist also finds it difficult to work with young clients. A case example is children which have autism or Asperger’s.

*“Adolescents with diagnoses such as autism*, *and Asperger’s*. *It’s a bit difficult to connect*. *When I’m asking*, *the answer is just confused and confusing*. *If you are offline*, *you can overcome it with tools such as drawing*, *they are usually okay*, *but if you are online*, *they don’t want to” (P2)*

Related to these difficulties, online sessions also means psychologist have limited working facilities to help the clients. This challenge exists in the assessment and intervention process. In the intervention process, media limitations for drawing or another activity that they can do together are not available. In the assessment process, psychologists only can rely on self-assessment and have difficulties using projective tests.

*“Giving intervention that needs media is quite challenging*. *Like using a jam board but there is an internet connection problem or drawing*. *It’s difficult because it should be on the application or clients don’t want to do it because it looks weird*, *A big enough challenge might be an assessment*, *a formal assessment using a test kit was a problem yesterday*, *because*, *for some of these disorders*, *we will be greatly helped by test kits*, *such as personality assessments*. *So*, *it’s not just a self-reported inventory*, *but a projective one*. *For example*, *TAT*, *Thematic Apperception Test*. *Because TAT in a face-to-face setting also takes a long time*. *It will help the client to be more open with his story*, *and more aware of his condition*. *It helps in mapping clients’ needs that have not been met so that conditions like this arise*, *what their needs are*, *and how to cope with the problem*. *Those are the tendencies*. *Now for online*, *it’s very difficult for us to use projective tests*, *or personality tests*, *so it’s only limited to self-assessment*.*” (P2)*

These difficulties then being categorized into three organizing themes which are: (1) difficult to work on moderate or severe psychological problems; (2) difficult to work on young clients; and (3) limited working facilities. These challenges and then can be categorized into perceived efficacy challenges.

#### Attitudes towards online sessions

The success of online mental health services needs cooperation between the psychologist and the clients. Online mental health services which consider new services in Indonesia make some psychologists are not really believed or have confidence enough that the sessions will work well for the clients because they do not have enough information and training to provide these sessions.

“At the beginning of the pandemic, I didn’t want to do online counseling because I realized that my capacity was still lacking in experience because when I was in college I never got special lessons to do online counseling” (P1)

Online mental health services also have to deal with clients’ cooperation. In some cases, usually for clients which use messenger, are in a hurry because of time limitations. Thus, they are just asking for a solution to their problems quickly and do not want to share more information with the psychologist.

*“If it’s counseling*, *the decision-making goes back to the client*. *We’re trying to give him an idea of your condition like this*, *you know now*, *we’re giving you risk factors*, *protective factors*. *But still*, *the decision-making will go back to him*. *I don’t know why*, *but so far*, *more people are online*, *they want to tell a little story*, *and get a lot of advice*, *and the advice is purely from us*, *they are the ones who carry it out*. *Maybe it’s because they think online is more concise*, *you can go anywhere*, *don’t meet up*, *don’t go out*. *Maybe it’s because of that*, *I don’t know why*. *So*, *when probing it still doesn’t tell much*. *That’s all I can tell you*, *then what should I do*, *just give me feedback now*. *It was many times and made uncomfortably*. *If it’s offline in an hour*, *I can dig deeper*, *so I can get more information” (P3)*

In some cases, psychologists also find that using online services is more comfortable and accessible. On the other hand, this easiness makes the clients get used to online sessions and do not want to do in-person sessions even though they need it as the advanced treatment from online sessions before.

*“I’m afraid that the client is already used to online sessions because it’s easy to access*. *Just in case he needs something offline one day*, *but because he’s used to doing online*, *it’s a bit of effort*. *For example*, *he experienced a traumatic event that has occurred in moderate conditions and requires offline intervention*, *but he is used to doing an online session*. *Well he might be thinking how come his problem can’t be handled” (P5)*

These challenges can be organized into three themes which are: (1) doubting whether the session will work; (2) depending on the client’s cooperation; and (3) dependency on online services. These themes then are categorized into challenges related to attitudes towards online sessions which need to be concerned in providing online sessions.

COVID-19 pandemic situation is one of the main factors of mental health services’ transformation into online services. Community’s need for online mental health services in the middle of pandemic conditions and the limitation of in-person services encourage the development of online mental health services in Indonesia. On the other hand, this change presents many challenges for online mental health providers. This research purpose is to map out the challenges that psychologist faces in providing online services.

Results of data analysis in this research have produced six major challenges faced by a psychologist who provides online mental health services in Indonesia. These challenges are: (1) building engagement difficulties (difficult to read client’s emotion, difficult to express feelings/empathy, difficult to engaged/connected with the client); (2) risk of getting distracted (internet and technical problems, risk of me getting distracted, risk of the client getting distracted during session); (3) maintain professional boundaries during the session (difficult to keep professional boundaries, difficult for the client to find a suitable space for online session, difficult for me to find a suitable space for the online session, scheduling); (4) keep the personal information and confidentiality of the sessions (confidentiality of the session, keep personal information); (5) perceived efficacy (difficult to work with moderate or severe psychological problems, difficult to work with young clients, emergency condition, limited working facility); (6) attitudes towards online sessions (doubting whether the session will works, depend on client’s cooperation, dependency with online services).

Interview questions for this research are developed from research by Békés et al. (2021) which maps out the challenges faced by a psychotherapist in providing online services through statistical research. According to this research, challenges faced by a psychotherapist is an emotional connection (feeling connected with patients, reading emotions, expressing or feeling empathy), distraction during sessions (therapist or patient), patients’ privacy (private space, confidentiality), and therapists’ boundaries (professional space, boundary setting) [6]. Similar to the results shown by that study, some of the challenges are also founded in this research.

Difficulties in building engagement challenges are related to reading clients’ emotions, expressing empathy, and being connected with the clients. Reading clients’ emotion is difficult because the psychologist only can hear the voice and even the client’s face is shown on the screen, no details like hands move are available. On the psychologist’s side, it is challenging to express empathy only through verbal. This is because some clients are using messenger for their sessions. The difficulties to read and express emotions then are leading to difficulties to be engaged or connected with the clients. These conditions are related to disengagement theory which says that online communication brings a negative effect on psychological well-being because it is time-consuming. Thus, online communication is considered a poor “virtual substitute” for in-person communication [13]. Therefore, an online session is a good option for providing mental health services during pandemic situations. If these difficulties in building a connection between psychologists and clients can be overcome, forming this therapeutic connection can predict a good outcome of the session [14].

The risk of getting distracted challenge is involving distraction from internet/technical problems and distraction because of surrounding conditions. Online sessions usually were held at home, both for the psychologist and the clients. Psychologist as professionals usually prepare a workroom that is conducive for an online session, but clients sometimes do not have private space and people around them has the possibility to distract the sessions. The transitions from in-person sessions into online sessions might cause more distractions because of some factors, such as popped-up notifications on the device, self-view in video conferencing, or fatigue because of the long online hours [15, 16].

Another challenge in providing online mental health services is the difficulties to maintain professional boundaries which are needed to keep professional even though there are problems like connection obstacles or improper conditions during the sessions. This maintaining professional boundaries challenge is related to the commitment to scheduling the sessions which involve the challenge to make the clients read the consent and stay at the sessions. Afterward, confidentiality and keeping personal information are other challenges faced by a psychologist in providing online sessions. This issue becomes a challenge because psychologists cannot control if there are other people around the clients. Sometimes online session also involves a staff responsible for the meeting, and difficult to handle the amount of online personal information shared between the therapist and clients.

Despite the four challenges mentioned before, there are perceived efficacy and attitudes towards online session challenges. Perceived efficacy is including challenges to working with moderate or severe psychological problems, working with young clients, and limited working facilities. Working together with children or adolescents are quite challenging because children/adolescent may think they do not need counselling or psychotherapy unlike their parents, children/adolescents may come with a completely different problem compared to the problems which have been noticed by their parents, and children/adolescent may do not notice what happened with them, even though they are realized about their problem they might not report their symptoms [17]. In this condition, psychologists need to find multiple sources for the assessment and treatment management which might be limited in the online sessions. Observations are limited, psychological tests are limited, and media to assess and work together are also limited. Overcoming this problem might be improving the use of online service sessions in the future.

The other challenges faced by a psychologist is an attitude toward online services which includes a belief that online services are working well with the clients and challenges related to the client’s cooperation in the sessions. Due to emergency conditions because of COVID-19 mental health services are going through a rapid change from in-person services to online sessions. As a result of these changes, some psychologists are having difficulties believing whether these online sessions are going to work well with the patients. In this condition, psychologist belief and attitudes are needed, because these beliefs will play a role to produce better changes in psychotherapy sessions. Trust between the psychologist and the clients will form a working alliance that ensures that the session will be successful [18, 19].

### Challenges related to local culture and situations

In addition to the findings that have been mentioned above, there are several challenging findings related to Indonesian culture in providing online mental health services. First, due to time limitations, many clients do not want to share more stories about themselves but seek much advice from the psychologist. Some of the clients which use messenger are typing their stories in a very short message then when the psychologist asked more explanations, clients do not want to share but ask for advice or solution directly from the psychologist. This attitudes also happen with the clients which use the other online media. Thus, some psychologists find it uncomfortable and disturb the session. Second, improper clothes from clients are distracting. Indonesians are very concerned about clothes when coming to attend a formal meeting. In online session cases, clients are wearing improper clothes for formal meetings such as pajamas, sleeping dresses, sleeveless, and short pants because most of the clients have the sessions at home. These kinds of clothes might be not a big deal in other countries, but Indonesians might consider them impolite and will distracting during the online sessions.

Besides cultural related challenges, some conditions in the present time also cause several challenges. COVID-19 challenges condition which makes people need to stay at home are causing stress not only for clients but also for psychologists. These conditions make them need to have more control, and more emotional regulation to work as a professional in online sessions. The other condition is some psychologist think that working with clients who have suicidal thoughts are very difficult in online sessions because of no integrated services available at this time. If something emergency happens to the clients, the psychologists need to work together with the family, search which institutions are related like hospitals or police stations which close to the clients, and contact these institutions with different hotlines. Psychologists are concerned if there is an integrated hotline that can be used in this kind of situation, it might be very helpful for supporting online mental health services.

### Implications, limitations, and future directions

Building engagement which is one of the challenges faced by psychologists including expressing empathy, reading clients’ emotions, and feeling connected with the clients is considered the core of the psychological sessions. Difficulties in this aspect may be overcome with training for the psychologist to handle online training. Thus, the psychologist can increase their trust in online services, build working alliances with the clients, manage these other challenges mentioned then reach the goal of the sessions. This training also maybe can increase positive attitudes towards online sessions. By increasing these positive attitudes, the effectiveness of online sessions can be experienced.

Besides providing training for the professional (psychologist), application usage for online sessions can be considered making improvements to manage these challenges. If using existing applications can cause some problems such as difficulties to keep confidentiality of the sessions and keep personal information, mental health providers can think about developing their application which opens the possibility to have full control of all of the information shared during the sessions.

Online mental health services are a great way to facilitate the provision of psychological services in this COVID-19 pandemic era. Mapping these challenges can bring a great contribution to the online mental health services development in Indonesia. If the challenges can be identified, further action can be taken to develop online mental health services which have started in Indonesia.

Indonesia is a big country, many areas in Indonesia still could not access any mental health services due to accessibility to the services and limited number of mental health practitioner available [20]. Therefore, it can be referred from the interviews that converting mental health services into online is a good start for future mental health service system development. Even though, the online services only available in big cities but people from all over Indonesia can access the service. Not limited only within the country, Indonesians which living abroad also can access this service. This is seen as more helpful way because accessing mental health service in the other country might still have barrier like language barrier and cultural differences.

However, the results of interviews with eight psychologists as professionals who provide online mental health services cannot be interpreted as representative of all online mental health services available in Indonesia. Eight participants which participated in this study were mostly the ages who still have the possibility to adapt fast to the technology but still remain a question if the professionals are not at those ages. Furthermore, the most psychologist who participated only has experience of fewer than 10 years, it might have differences from a psychologist who has more than 10 years of clinical experience.

Future studies might be considering doing a quantitative approach to these aspects founded. Thus, more professionals can be involved and can establish challenges that psychologist faces more generally. Future studies also can try to map these challenges in online mental health services from different points of view, such as mental health provider’s institutions, clinical supervisors, or clients.

Due to the importance and great innovation of online mental health services which facilitate the provision of psychological services, these results are expected to be useful in the future. These challenges which are mapped out as the results of this research, can be used as considerations for online mental health providers, both policy maker, professional, institutions, and related organizations in Indonesia. This research also can be used for references to other developing country which has similar situation with Indonesia, such as limited services available, no integrated service available, and experience mental health system change because of COVID-19 outbreak. In the future, online mental health services can be used by every people with different psychological problems with different problems as well as in-person therapy in the future, both in COVID-19 situation and any situation.

## Conclusions

Six challenges in providing online mental health services were founded: (1) building engagement difficulties; (2) risk of getting distracted; (3) maintaining professional boundaries during the session; (4) keep the personal information and confidentiality of the sessions; (5) perceived efficacy; (6) attitudes towards online sessions. Perceived efficacy and attitudes towards online sessions are the new findings found in this study. In addition, some cultural and condition-related issues are also mapped out. These cultural-related challenges such as clients’ demands and improper clothes, then pandemic conditions, and integrated hotlines are challenges related to the current situation in Indonesia. Addressing the unmet needs described in this study would help mental health service development in Indonesia and provide information for other developing country to improve mental health service systems, both in COVID-19 pandemic situation and in the future.

## Supporting information

S1 Appendix PLOS questionnaire on inclusivity in global research. (PDF)

S2 Appendix Minimal anonymized data set. (PDF)

## References

[1] The Jakarta Post. Regions close schools, cancel public events because of COVID-19 [Internet]. The Jakarta Post. 2020 [cited 2022 Mar 8]. Available from: https://www.thejakartapost.com/news/2020/03/15/regions-close-schools-cancel-public-events-because-of-covid-19.html

[2] Alexander MO Benjamin Roberts, Narnia Bohler Muller & Kate. Coronavirus: Lockdown Op-ed: The hidden struggle: The mental health effects of the Covid-19 lockdown in South Africa. Daily Maverick [Internet]. 2020 May 13 [cited 2023 Jan 25]; Available from: https://www.dailymaverick.co.za/article/2020-05-13-the-hidden-struggle-the-mental-health-effects-of-the-covid-19-lockdown-in-south-africa/

[3] Saptandari EW. Covid-19 and Mental Health: The Growing Need of Telecounseling in Indonesia. Bul Psikol. 2020 Dec 23;28(2):99–112.

[4] Serafini G, Parmigiani B, Amerio A, Aguglia A, Sher L, Amore M. The psychological impact of COVID-19 on the mental health in the general population. QJM Int J Med. 2020 Aug 1;113(8):531–7. doi: 10.1093/qjmed/hcaa201 32569360 PMC7337855

[5] Situmorang DDB. Online/Cyber Counseling Services in the COVID-19 Outbreak: Are They Really New? J Pastor Care Couns. 2020 Oct 1;74(3):166–74. doi: 10.1177/1542305020948170 32967547 PMC7528539

[6] Békés V, Aafjes-van Doorn K, Luo X, Prout TA, Hoffman L. Psychotherapists’ Challenges With Online Therapy During COVID-19: Concerns About Connectedness Predict Therapists’ Negative View of Online Therapy and Its Perceived Efficacy Over Time. Front Psychol [Internet]. 2021 [cited 2022 Mar 8];12. Available from: https://www.frontiersin.org/article/10.3389/fpsyg.2021.705699 doi: 10.3389/fpsyg.2021.705699 34367030 PMC8339462

[7] Institute of Medicine. Mental Health Services in General Health Care: A Conference Report, Volume I [Internet]. Washington, DC: The National Academies Press; 1979 [cited 2022 Mar 15]. 294 p. Available from: https://www.nap.edu/catalog/9935/mental-health-services-in-general-health-care-a-conference-report

[8] Ifdil I, Ardi Z. Konseling Online Sebagai Salah Satu Bentuk Pelayanan E-konseling. J Konseling Dan Pendidik. 2013 Feb 28;1(1):15–22.

[9] Indonesian Ministry of Women Empowerment and Child Protection. Layanan SEJIWA Lindungi Kesehatan Mental Masyarakat di Masa Pandemi Covid-19 [Internet]. Kementerian Pemberdayaan Perempuan dan Perlindungan Anak. 2021 [cited 2022 Mar 8]. Available from: https://www.kemenpppa.go.id/index.php/page/read/29/3178/layanan-sejiwa-lindungi-kesehatan-mental-masyarakat-di-masa-pandemi-covid-19

[10] CNN Indonesia. Aplikasi Konsultasi Kesehatan Jiwa Anti-Cemas Online [Internet]. teknologi. 2020 [cited 2022 Mar 8]. Available from: https://www.cnnindonesia.com/teknologi/20200429192710-185-498634/aplikasi-konsultasi-kesehatan-jiwa-anti-cemas-online

[11] Boblin SL, Ireland S, Kirkpatrick H, Robertson K. Using Stake’s Qualitative Case Study Approach to Explore Implementation of Evidence-Based Practice. Qual Health Res. 2013 Sep;23(9):1267–75. doi: 10.1177/1049732313502128 23925405

[12] Attride-Stirling J. Thematic networks: an analytic tool for qualitative research. Qual Res. 2001 Dec;1(3):385–405.

[13] Manfrida G, Albertini V, Eisenberg E. Connected: Recommendations and Techniques in Order to Employ Internet Tools for the Enhancement of Online Therapeutic Relationships. Experiences from Italy. Contemp Fam Ther. 2017;39(4):314–28. 29200614 10.1007/s10591-017-9439-5 PMC5686290

[14] Flückiger C, Del Re AC, Wampold BE, Horvath AO. The alliance in adult psychotherapy: A meta-analytic synthesis. Psychotherapy. 2018 Dec;55(4):316–40. doi: 10.1037/pst0000172 29792475

[15] Bailenson JN. Nonverbal Overload: A Theoretical Argument for the Causes of Zoom Fatigue. Technol Mind Behav [Internet]. 2021 Feb 23 [cited 2022 May 3];2(1). Available from: https://tmb.apaopen.org/pub/nonverbal-overload/release/2

[16] Brainard R, Watson L. Zoom in the Classroom: Transforming Traditional Teaching to Incorporate Real-Time Distance Learning in a Face-to-Face Graduate Physiology Course. FASEB J. 2020;34(S1):1–1.

[17] Bhide A, Chakraborty K. General Principles for Psychotherapeutic Interventions in Children and Adolescents. Indian J Psychiatry. 2020 Jan;62(Suppl 2):S299–318. doi: 10.4103/psychiatry.IndianJPsychiatry_811_19 32055072 PMC7001347

[18] Sandell R, Lazar A, Grant J, Carlsson J, Schubert J, Broberg J. Therapist attitudes and patient outcomes: II. Therapist attitudes influence change during treatment. Psychother Res. 2007 Mar;17(2):196–204.

[19] Wampold BE. How important are the common factors in psychotherapy? An update. World Psychiatry. 2015 Oct;14(3):270–7. doi: 10.1002/wps.20238 26407772 PMC4592639

[20] Putri AK, Gustriawanto N, Rahapsari S, Sholikhah AR, Prabaswara S, Kusumawardhani AC, et al. Exploring the perceived challenges and support needs of Indonesian mental health stakeholders: a qualitative study. Int J Ment Health Syst. 2021 Nov 8;15(1):81. doi: 10.1186/s13033-021-00504-9 34749767 PMC8573764

