# Supplementary material for: Online mental health services during COVID-19 pandemic in Indonesia: Challenges from psychologist perspective
Source: PLoS One. 2023 Jun 23;18(6):e0285490. doi: 10.1371/journal.pone.0285490 (PMC10289369; doi:10.1371/journal.pone.0285490)
Supplement: S1 Appendix — (PDF) [file pone.0285490.s001.pdf]

WORDS	THEMES	ORGANIZED THEMES
In the process of building rapport, it is much more difficult, especially in the form of a phone call that cannot be observed, it means we only focus on the voice, story, and intonation, which means that understanding people's information is much more difficult than I met them in person	Difficulties in reading emotions	Building engagement difficulties
The observations are only the face, no hands, no legs, and nobody posture. Because it's just his face. If we are offline, we can know, for example, if there is an indication of anxiety, the hand must be doing something. His hands were either clutching the table or the pillow, or the shirt. We can't observe that. We don't know whether his legs are moving. Or squeezing for people who are holding back the story, their hands are really clenched into fists. Squeezing his clothes for example	Difficulties in reading emotions	
The other obstacle is less effective in comforting her. Usually, I can show gestures that show empathy, so she's more comfortable, for example, if a woman, I can hold her hand, I can pat her, that's usually comforting for the client. But if you're online, you can't do things like that. It's just voice and it's also limited for comforting them	Difficulties to express feelings/emotions	
When analyzing the problem, we can't help but generalize the client's problems, it should be personalized, but because of this short rapport building we don't really explore the client's character, in the end, we just focus on the problem, and how to solve it. The building rapport is just left as it is	Difficulties to feel engaged/connected with the client	

Because this is online there is latency, yes connection problems, but this internet problem is still a pretty important problem. Inevitably it will affect how the session goes and it feels comfortable for the client because there is a lot of feedback from the client, some say there was a connection problem earlier so there are some things I have to make sure again I have to ask the psychologist again, or they say the psychologist video is a bit lagging. It's really disturbing because the emotions are already established, the client is open, suddenly it's “deg” (stop) like that, and sometimes the client is thrown off from the platform because of the connection. When he got the right moment, he was able to release his emotions, he was also in the phase to start a discussion, suddenly he is out when he came back the moment was gone, it was very distracting	Internet and technical problem	Risk of getting distracted
There are some people who take place in the cafe, it's automatically noisy right, I'm disturbed too, right, I'm still focused, I need to take care of their feelings, I try to be careful with my sentences while the noise is really annoying. We can only remind them	Risk of me getting distracted	
I used to give her relaxation because she really needed it but suddenly her mom came in and she opened her eyes "Wait a minute, Mom! Close again!” even though it was a very deep session. That’s very annoying	Risk of the clients getting distracted	
Honestly, for me personally online is more tiring, maybe one of them because before starting an online session, there was a concern about internet problems, either on my internet or the client’s internet. It means that if the session is online, there are clients from remote areas as well, whose internet stability is not good. And we can't say sorry, Ms. the network is having problems, even though we are the ones who are upset that the network is bad. So sometimes being patient with technical things is tiring	Difficulties to keep professional boundaries	Maintaining professional boundaries during the sessions
It sometimes happens in the beginning, counseling in a public place. And I need to control my emotions because we are still focused and there are noisy people which are very annoying. While I input information and I have to verbalize it again and I need concentration to organize it	Difficult for the clients to find a suitable space for the online session	

In my house house there is no wifi, so when it was raining, the connection was disconnected. That's will impact the sesstion. Sometimes to log in again I need to wait for ling time, I want to make sure my connection first. Not comfortable for sure	Difficult for me to find a suitable space for the online session	
We always remind that counseling sessions must be followed properly, some clients are counseling while lying down, rolling around, smoking cigarettes, and some are eating and holding a spoon. There is a client who does the counseling while in the kitchen peeling onions. There is someone lying on the bed, her cellphone is lifted, and the camera is moving here and there. This distracts me, the session also becomes ineffective because looking at the screen rotates things, it's making me dizzy. Difficult to condition. And when I remind her, she sat but lay down again. Sometimes their clothes are not proper, it’s too revealing. It's indeed at home but that doesn't mean you wear clothes for sleeping. I’m thinking can this session be serious for discussing the problem	Scheduling is difficult	
Usually, the client using chat is a bit hectic because the time is limited to one hour, one and a half, or two hours. Well, usually someone typed a long story straight away, so the psychologist needs to understand, right, read it first, digest it first, and complaints from the long story sometimes when we finish and give a response the client doesn't respond anymore, doesn't reply	Scheduling is difficult	
If it's me, I'll take good care of the data, but if he's talking about his mother during the session and his mother can hear it, I don't know, I can't control it either. It's more conditioned if it's offline	Confidentiality of the online session	Keeping personal information and confidentiality of the session

Application for video conferencing we used Zoom. Sometimes if there's something wrong, the admin staff has to be on standby, so there are 3 people involved, right. So, if there's anything and the admin is not on standby, it will be stuck. And the psychologist doesn’t have the client's numbers because it will be handled by the office, so there is really a third party. If one day there is a system error, we have to contact the IT person as the admin. Well, like it or not, the IT person will know about client data. So, in the clinical setting, many people are involved and those are not psychological people. So much riskier	Difficulty to keep my and clients' personal information	
If we go online, our names will be listed on Zoom. Well, one day I saw on my “linked in that my profile was seen by my client. So, it's more accessible, right? If you're in the room, just your full name and nickname, but what they remember is just your nickname. But yes, some people from online session saw my linked-in, my Instagram	difficulty to keep my and clients' personal information	
Adolescents with diagnoses such as autism, and Asperger's. It's a bit difficult to connect. When I’m asking, the answer is just confused and confusing. If you are offline, you can overcome it with tools such as drawing, they are usually okay, but if you are online, they don't want to	Difficult to work on moderate or severe psychological problems	Perceived efficacy
For youth clients under 18 years. Adolescents have various issues, because sometimes their families are the one who notice and they bring their children to the counselling session. They might be confused, so sometimes their expressions are flat and the information is very difficult to get. What's the purpose of coming, I don't know, I was told. Even though students studying from home can be a problem because there are lots of stressful assignments, cabin fever can't meet their friends, they feel alone, when they are in a period of searching for their identity, it's hampered because everything is online. So they are not comfortable. In fact, many of them just after a few sessions that the story just came out. But for adults, they are already aware and have the intention to come, so yes, they can start right away	Difficult to work on young clients	

Giving intervention that needs media is quite challenging. Like using a jam board but there is an internet connection problem or drawing. It’s difficult because it should be on the application or clients don’t want to do it because it looks weird, A big enough challenge might be an assessment, a formal assessment using a test kit was a problem yesterday, because, for some of these disorders, we will be greatly helped by test kits, such as personality assessments. So, it's not just a self-reported inventory, but a projective one. For example, TAT, Thematic Apperception Test. Because TAT in a face-to-face setting also takes a long time. It will help the client to be more open with his story, and more aware of his condition. It helps in mapping clients' needs that have not been met so that conditions like this arise, what their needs are, and how to cope with the problem. Those are the tendencies. Now for online, it's very difficult for us to use projective tests, or personality tests, so it's only limited to self-assessment	Limited working facilities	
At the beginning of the pandemic, I didn't want to do online counseling because I realized that my capacity was still lacking in experience because when I was in college I never got special lessons to do online counseling	Doubting whether the session will work	Attitudes towards online sessions
If it's counseling, the decision-making goes back to the client. We're trying to give him an idea of your condition like this, you know now, we're giving you risk factors, protective factors. But still, the decision-making will go back to him. I don't know why, but so far, more people are online, they want to tell a little story, and get a lot of advice, and the advice is purely from us, they are the ones who carry it out. Maybe it's because they think online is more concise, you can go anywhere, don't meet up, don't go out. Maybe it's because of that, I don't know why. So, when probing it still doesn't tell much. That's all I can tell you, then what should I do, just give me feedback now. It was many times and made uncomfortably. If it's offline in an hour, I can dig deeper, so I can get more information	Depending on the client’s cooperation	

I'm afraid that the client is already used to online sessions because it's easy to access. Just in case he needs something offline one day, but because he's used to doing online, it's a bit of effort. For example, he experienced a traumatic event that has occurred in moderate conditions and requires offline intervention, but he is used to doing an online session. Well he might be thinking how come his problem can't be handled	Dependency on online services
